# Elevated IgG and IgM Autoantibodies to Advanced Glycation End Products of Vascular Elastin in Hypertensive Patients with Type 2 Diabetes: Relevance to Disease Initiation and Progression

**DOI:** 10.3390/pathophysiology29030034

**Published:** 2022-08-01

**Authors:** Krasimir Kostov, Alexander Blazhev

**Affiliations:** 1 Department of Pathophysiology, Medical University-Pleven, 1 Kliment Ohridski Str., 5800 Pleven, Bulgaria; 2 Department of Biology, Medical University-Pleven, 1 Kliment Ohridski Str., 5800 Pleven, Bulgaria; yalishanda9@gmail.com

**Keywords:** hypertension, type 2 diabetes, advanced glycation end products (AGEs), autoantibodies to AGEs of vascular elastin

## Abstract

The increased glycation of elastin is an important factor in vascular changes in diabetes. Using the ELISA method, we determined serum levels of IgM and IgG autoantibodies to advanced glycation end products of vascular elastin (anti-AGE EL IgM and anti-AGE EL IgG) in 59 hypertensive patients with type 2 diabetes (T2D) and 20 healthy controls. Serum levels of matrix metalloproteinases-2 and -9 (MMP-2 and MMP-9) and the C-reactive protein (CRP) were also determined. The levels of anti-AGE EL IgM antibodies in the T2D group were similar to those in the control group, while those of anti-AGE EL IgG antibodies were significantly higher (*p* = 0.017). Significant positive correlations were found between the levels of anti-AGE EL IgM antibodies and MMP-2 (*r* = 0.322; *p* = 0.013) and between the levels of anti-AGE EL IgG antibodies and CRP (*r* = 0.265; *p* = 0.042). Our study showed that elevated anti-AGE EL IgG antibody levels may be an indicator of the enhanced AGE-modification and inflammatory-mediated destruction of vascular elastin in hypertensive patients with T2D. Anti-AGE EL IgM antibodies may reflect changes in vascular MMP-2 activity, and their elevated levels may be a sign of early vascular damage.

## 1. Introduction

Diabetes mellitus is a chronic disease with an increasing frequency over the last decade [1], with type 2 diabetes (T2D) accounting for more than 90% of all diagnosed cases [2]. In the long term, patients with T2D are at increased risk of developing cardiovascular disease (CVD), and the identification of specific biomarkers may improve their treatment [3]. One group of biomarkers that can be used are the autoantibodies to advanced glycation end products (AGEs) [4,5].

AGEs are formed by non-enzymatic reactions between the carbonyl groups of reducing sugars, such as glucose, and the free amino groups of a number biomolecules in the body, via the Maillard reaction [6]. This reaction is followed by the generation of a reversible Schiff-base adduct, which rearranges into a more stable and covalently bonded Amadori product. The Amadori product then undergoes irreversible chemical modifications that generate AGEs [7]. The glycation process can affect all proteins in the body, including circulating, extracellular, and intracellular proteins, such as hemoglobin, albumin, insulin, immunoglobulins, low-density lipoproteins, lens crystalline proteins, collagen (COL), and elastin (EL) [8,9,10]. Other biomolecules, such as lipids and DNA, can also be modified in a similar way [11]. Particularly vulnerable to glycation are long-lived molecules such as COL and EL in the vascular extracellular matrix (ECM), due to the slow rate of their turnover [12,13]. In diabetes, AGEs can also be formed through the polyol pathway, where intermediates are even more potent glycation agents than glucose [14,15]. The non-enzymatic glycation of biomolecules is accelerated in patients with diabetes, but also occurs in non-diabetic subjects [16].

EL is the main structural element of the arteries and has the lowest turnover rate of all components of vascular ECM (half-life of about 40 years) [17]. Its mechanical properties are crucial for normal arterial function, and for this reason, it is widely involved in the pathogenesis of CVD [18]. Biochemical analyses showed that after only twelve days of incubation at a sugar concentration of 250 mmol/L, one of the five available lysines per elastin monomer was already glycated. At longer incubation times, the generation of AGEs increases, which can significantly alter the physical properties of EL [19]. Changes in vascular EL in diabetes and the formation of cross-links with AGEs may contribute to its fragility and fragmentation, which may be amplified by concomitant hypertension [20].

Structural changes in biomolecules due to AGE modifications are associated with the formation of new epitopes that make them potential targets of the immune system. Anti-AGE antibodies that can be used as a biomarker for vascular damage have been found in the sera of patients with diabetes [4,21,22,23]. Due to their immunogenicity, AGEs can cause inflammation by stimulating the AGE receptor (RAGE), which triggers a series of signaling cascades and activates pro-inflammatory genes [24,25,26]. Inflammation, in turn, may enhance the activity of matrix metalloproteinases (MMPs) in the vascular wall [20].

In our study, we used as an antigen human aortic α-elastin, glycated in vitro, to determine the serum levels of IgM and IgG autoantibodies to AGEs of vascular elastin (anti-AGE EL IgM antibodies and anti-AGE EL IgG antibodies) in hypertensive patients with T2D. We also measured serum levels of MMP-2, MMP-9, and the C-reactive protein (CRP) as indirect biomarkers for elastase activity and low-grade systemic inflammation.

## 2. Materials and Methods

### 2.1. Screening of the Patients and Controls

The studied clinical contingent includes patients with T2D who were admitted for periodic control and monitoring at the Dr. Georgi Stranski University Hospital in Pleven. Control subjects were clinically healthy age-matched volunteers. The patients and controls were screened for hypertension according to the 2018 ESC/ESH Clinical Practice Guidelines. Blood pressure (BP) was measured on the left arm in a sitting position after 5–10 min of rest. Hypertension was defined as systolic BP ≥ 140 mmHg and/or diastolic BP ≥ 90 mmHg, or if the patients had been diagnosed or had taken antihypertensive drugs at any time during the preceding six months. Normal BP was defined as systolic BP 120–129 mmHg and diastolic BP 80–84 mmHg.

### 2.2. Immunological and Biochemical Assays

To measure the levels of anti-AGE EL IgM and anti-AGE EL IgG antibodies and the other laboratory parameters, blood was drawn into vacutainer tubes and was centrifuged at 2500 rpm for 10 min to separate the serum. Biochemical analyzes were performed immediately, and serum samples for the immunoassay were stored at −70 °C until testing.

#### 2.2.1. Determination of Anti-AGE EL IgM and Anti-AGE EL IgG Antibodies

AGE-elastin was obtained via the incubation of human aortic α-elastin (1.33 mg/mL) with 100 mmol/L glucose for 30 days, as described by Baydanoff et al. [27]. A blocking ELISA was used for the detection of IgM and IgG autoantibodies to AGEs of vascular EL. The 96-well plates were coated with AGE-elastin (5 μg/mL) and incubated with 100 μL of human sera (diluted 1:20) for 1 h at 37 °C. Then, 100 µL of goat anti-human IgM Ab, Fc5µ, HRP conjugate (AP114P, Sigma-Aldrich, St. Louis, MO, USA) and goat anti-human IgG Ab, Fc, HRP conjugate (AP113P, SigmaAldrich, St. Louis, MO, USA), respectively, were added to each well. Immunoconjugates were diluted 1:10,000 and ortho-phenylenediamine was used as the chromogen. The reaction was stopped by adding 50 μL/well of sulfuric acid (4 M H_2_SO_4_), and the optical density was measured on a Coulter Microplate Reader UV Max (Molecular Devices Corp., Menlo Park, CA, USA) at a wavelength of 492 nm. All samples were tested in triplicate.

#### 2.2.2. Determination of MMP-2 and MMP-9

Serum levels of MMP-2 and MMP-9 were determined by ELISA kits from R&D Systems (MMP-2, cat. no. DMP2F0 and MMP-9, cat. no. DMP900). The samples were analyzed on a Coulter Microplate Reader UV Max at a wavelength of 450 nm.

#### 2.2.3. Biochemical Analysis

Serum CRP levels were measured by particle enhanced turbidimetry. Glycated haemoglobin (HbA1c) levels were determined by a turbidimetric inhibition immunoassay. Total cholesterol (TC), low-density lipoprotein cholesterol (LDL-C), high-density lipoprotein cholesterol (HDL-C), and triglyceride (TG) were measured by enzymatic methods. All samples were analyzed on a Cobas Integra 400 system (Roche Diagnostics, Basel, Switzerland).

### 2.3. Statistical Analysis

Statistical analyses were performed using IBM SPSS Statistics version 23.0 software (SPSS, Inc., Chicago, IL, USA). The differences between the means of two groups were assessed by an unpaired Student’s *t*-test. Correlation analysis was performed with Pearson’s correlation test. *p* values of less than 0.05 were considered statistically significant.

## 3. Results

### 3.1. Characteristics of the Study Population

The study population consisted of 59 hypertensive patients with T2D (age 60.8 ± 14.7 years; mean disease duration of 10.1 ± 7.8 years) and 20 healthy controls (mean age 61.5 ± 11.4 years). The clinical characteristics of the groups are shown in Table 1.

### 3.2. Comparison of Anti-AGE EL Antibody Levels between the T2D Group and Controls

**Figure 1:**
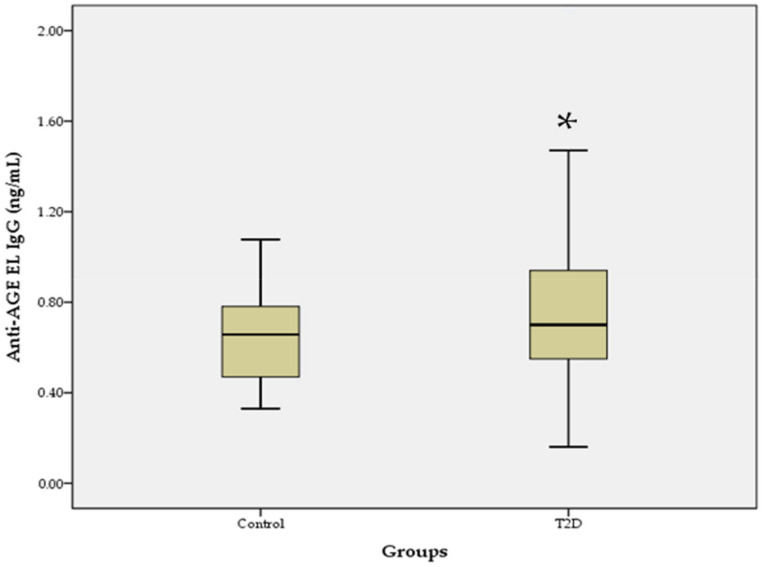
Serum levels of anti-AGE EL IgG antibodies in the T2D group compared to the control group. Data are represented as mean ± SD. * *p* < 0.05.

The levels of anti-AGE EL IgM antibodies in the T2D group were similar to those in the control group, and the difference was not statistically significant (0.46 ± 0.18 vs. 0.45 ± 0.13; *p* = 0.923). In contrast, the levels of anti-AGE EL IgG antibodies were significantly higher in the T2D group than in the control group (0.84 ± 0.48 vs. 0.65 ± 0.20; *p* = 0.017; Figure 1).

### 3.3. Correlation between Anti-AGE EL Antibody Levels and Clinical Features

**Figure 2:**
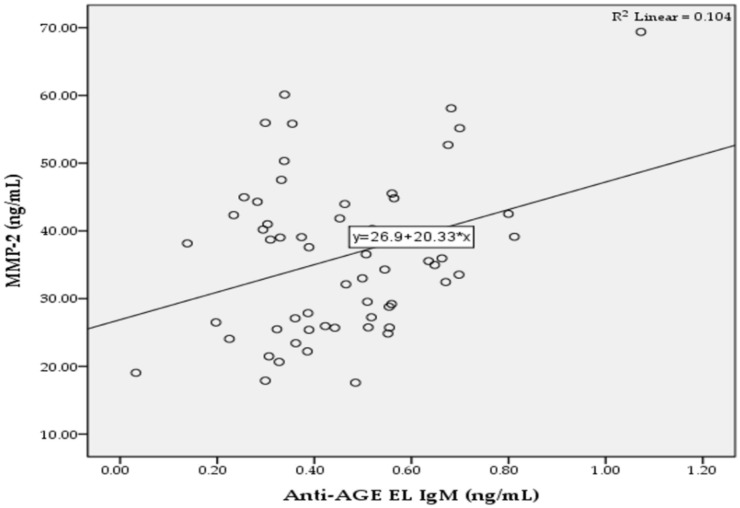
Correlation between the serum levels of anti-AGE EL IgM antibodies and MMP-2 in the T2D group.

**Figure 3:**
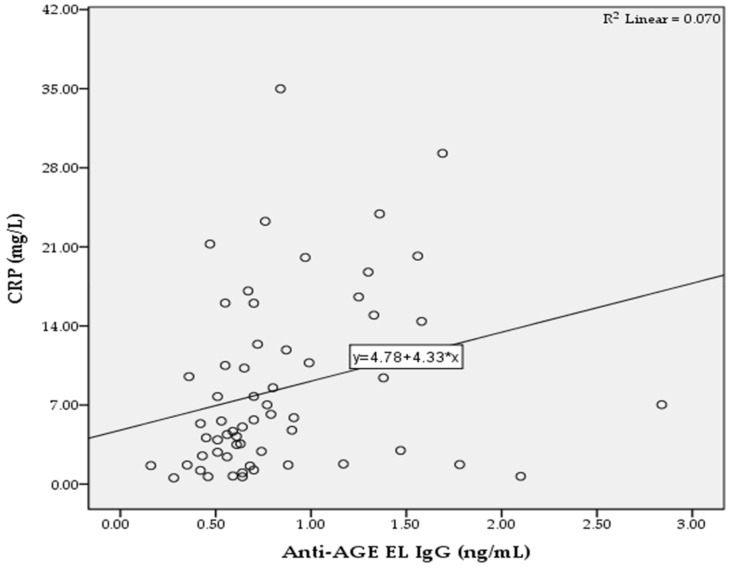
Correlation between the serum levels of anti-AGE EL IgG antibodies and CRP in the T2D group.

In the T2D group, we found significant positive correlations between the levels of anti-AGE EL IgM antibodies and MMP-2 (*r* = 0.322; *p* = 0.013; Figure 2), as well as between the levels of anti-AGE EL IgG antibodies and CRP (*r* = 0.265; *p* = 0.042; Figure 3).

## 4. Discussion

Autoimmunity is considered the major factor in the pathogenesis of type 1 diabetes (T1D), but also plays a role in T2D. A hallmark of autoimmune involvement in T2D is the presence of circulating autoantibodies [28]. The detection of elevated levels of autoantibodies to AGEs in patients with T2D raises the question of their role in the pathophysiology of the disease. Nikolov et al. have found that serum levels of total anti-AGE antibodies were significantly higher in hypertensive patients with T2D with microvascular complications than healthy controls and patients without such complications [22]. Similar results have also been reported in patients with T1D [29,30].

The accumulation of AGEs on long-lived proteins of the vascular tissue is closely related to the development of diabetic vascular complications, which makes the measurement of serum levels of total and class-specific autoantibodies to AGE EL important for the assessment of increased vascular risk in patients with T2D. Because elastin is a main structural element of arteries and is a potential target for the formation of AGEs [31], we investigated serum levels of IgM and IgG autoantibodies to AGEs of vascular EL (AGE EL) in patients with advanced T2D and hypertension, who are at increased cardiovascular risk. The results showed that the levels of anti-AGE EL IgG antibodies were significantly higher in the T2D group compared to the control group, while the levels of anti-AGE EL IgM antibodies were similar to those in the controls (Figure 1). The non-enzymatic glycation of vascular EL is a spontaneous process [32] that leads to the formation of autoantibodies against epitopes of AGE EL [29], both in normal aging and in diabetes [33]. The IgM class of autoantibodies predominates in the early stage of the immune response and later undergoes switching to the IgG class, which has the same antigen specificity [34]. Because T2D is a chronic disease, the levels of anti-AGE EL IgG antibodies are significantly higher than those of anti-AGE EL IgM antibodies compared to the controls. We also found a positive correlation between the serum levels of anti-AGE EL IgM antibodies and MMP-2, suggesting that these antibodies may serve as a biomarker for vascular damage in T2D (Figure 2). MMP-2 is an important ECM enzyme that can break down various substrates, such as COL, EL, fibronectin, and laminin [35]. A number of studies have shown that the dysregulation of MMP-2 may contribute to the development of diabetic vascular complications [36,37,38]. Elastases include five MMPs (MMP-2, -7, -9, -12, and-14), and serine and cysteine proteinases. They can cleave EL, leading to the formation of EL-derived peptides. Interestingly, these peptides are able to promote insulin resistance and the appearance of characteristic features of T2D, as well as to promote atherogenesis [39]. They can also promote angiogenesis, cell adhesion, proliferation, chemotaxis, protease activity, and apoptosis [40]. Our results showed that serum levels of MMP-2 and MMP-9 were significantly higher in the T2D group than in the control group, which may be an indirect sign of increased EL destruction in the arterial wall (Table 1).

The increased accumulation of AGEs in diabetic vascular tissue causes an inflammatory response characterized by leukocyte activation and the release of proinflammatory cytokines such as interleukin-1 (IL-1), interleukin-6 (IL-6), and tumor necrosis factor-α [41,42,43]. In response to these cytokines, the liver produces CRP, which is considered an important biomarker for systemic inflammation. IL-6 is the major inducer of CRP gene expression, with IL-1 potentiating this effect [44]. Our data show that there is a significant positive correlation between the levels of anti-AGE EL IgG antibodies and CRP as a marker for systemic inflammation (Figure 3). Therefore, this correlation suggests the presence of a direct relationship between the degree of inflammatory response and the levels of anti-AGE EL IgG antibodies. Our data also show that CRP levels were significantly higher in the T2D group compared to the control group (Table 1). Pickup et al. reported that IL-6 and CRP were elevated in the serum of patients with non-insulin-dependent diabetes mellitus [45]. In addition, elevated serum concentrations of AGEs in patients with T2D are an independent determinant of CRP levels [46]. CRP causes numerous proinflammatory and proatherogenic effects in endothelial cells, such as the decreased production of nitric oxide and prostacyclin, increased production of endothelin-1, and increased expression of adhesion molecules, monocyte chemotactic protein-1, interleukin-8, and plasminogen activator inhibitor-1 [47].

The exact role of anti-AGE antibodies in the pathophysiology of diabetes is not fully understood. It is thought that they may be part of a defense mechanism that serves to remove damaged or dysfunctional proteins as a result of enhanced AGE modifications [48]. In this regard, the likely role of anti-AGE EL IgM and anti-AGE EL IgG antibodies is that they may be involved in the removal of damaged glycated vascular EL and its metabolites through the formation of circulating immune complexes and their subsequent elimination by a mononuclear phagocytic system. An additional mechanism of complement activation and K-cell-mediated antibody-dependent cytotoxicity may contribute to the further destruction of EL in the arterial wall, and specific T- and B-lymphocytes may also be involved in this process [49,50,51].

A limitation of the study is the relatively small number of individuals studied, which requires these results to be confirmed in a larger cohort.

## 5. Conclusions

Our results showed that the levels of anti-AGE EL IgG antibodies were significantly higher in the T2D group compared to the control group, which can be explained by the chronic course of the disease. A positive correlation was found between the levels of anti-AGE EL IgG antibodies and CRP, suggesting a direct relationship between the levels of these antibodies and the grade of systemic inflammation in T2D patients. The levels of anti-AGE EL IgM antibodies may predominate in the early stages of the immune response, and the existence of a positive correlation between them and MMP-2 suggests that they may serve as predictors of early vascular damage. Therefore, it can be concluded that the measurement of serum levels of class-specific autoantibodies against AGE EL may be important for the overall assessment of vascular risk in T2D patients.

## Figures and Tables

**Table 1:** Clinical characteristics of the groups.

Variables	Healthy Control Subjects	Patients with T2D
	(*n* = 20)	(*n* = 59)
Sex, Male/Female	10/10	25/34
Age, years ^1^	61.5 ± 11.4	60.8 ± 14.7
Duration of T2D ^1^	N/A	10.1 ± 7.8
SBP, mmHg ^1^	121.5 ± 8.6	149.2 ±16.7 ***
DBP, mmHg ^1^	78.2 ± 7.5	83.0 ± 10.4
BMI, kg/m^2 1^	24.9 ± 2.4	28.4 ± 4.5 ***
HbA1c (%) ^1^	N/A	7.5 ± 1.8
TC, mmol/L ^1^	4.2 ± 0.7	5.2 ± 1.8 *
LDL-C, mmol/L ^1^	2.8 ± 0.8	3.0 ± 1.1
HDL-C, mmol/L ^1^	1.2 ± 0.2	1.0 ± 0.3 ***
TG, mmol/L ^1^	1.4 ± 0.4	2.7 ± 3.0
CRP, mg/L ^1^	1.1 ± 0.9	8.4 ± 7.9 ***
MMP-2, ng/mL ^1^	30.6 ± 1.8	36.2 ± 1.5 *
MMP-9, ng/mL ^1^	25.8 ± 2.8	38.4 ± 2.6 **

* *p* < 0.05, ** *p* < 0.01, *** *p* < 0.001; ^1^ Mean ± SD; N/A, not available; SBP, systolic blood pressure; DBP, diastolic blood pressure; BMI, body mass index; TC, total cholesterol; LDL–C, low-density lipoprotein cholesterol; HDL–C, high-density lipoprotein cholesterol; TG, triglyceride; CRP, C-reactive protein; MMP-2, matrix metalloproteinase-2; MMP-9, matrix metalloproteinase-9.

## Data Availability

The authors confirm that the data supporting the findings of this report are available within the article.
